# Obesity and Asthma in Children—Coexistence or Pathophysiological Connections?

**DOI:** 10.3390/biomedicines13051114

**Published:** 2025-05-04

**Authors:** Clarissa Mazzotta, László Barkai

**Affiliations:** 1 Azienda Sanitaria Locale della Provincia di Lecce, 73100 Lecce, Italy; 2 Department of Paediatrics and Adolescent Medicine, Faculty of Medicine, Pavol Jozef Šafárik University, 04001 Kosice, Slovakia; 3 Physiological Controls Research Center, University Research and Innovation Center, Obuda University, 1034 Budapest, Hungary

**Keywords:** asthma, obesity, pathophysiological mechanisms

## Abstract

The aim of this narrative review is to explore possible connections that might lead to both obesity and asthma; it will explain factors and mechanisms involved in disease pathogenesis, focusing particularly on diet and nutrients, the microbiome, inflammatory and metabolic dysregulation, lung function, the genetics/genomics of obese asthma, risk of exacerbation, atopy, and response to treatment. It highlights the role that obesity plays as a risk factor for and disease modifier of asthma, understanding the evidence supporting lifestyle changes in influencing disease progression. Pathophysiological mechanisms in obesity-related asthma have influences on the course of disease pathology. Due to these factors, the child with obese asthma needs a specific therapeutic approach taking into account the common unresponsiveness to bronchodilators, increased requirements for controller medications, poorer steroid effectiveness, and better response to leukotriene receptor (LTR) inhibitors. Therapeutic strategies centered on prevention are suggested and the development of resources to assist families with weight loss strategies and lifestyle changes is shown to be useful for effective weight control and optimal asthma management. Obese children with asthma generally should receive interventions that encourage daily physical activity, weight loss, and normalization of nutrient levels, and monitoring of common obesity-related sequelae should be considered by healthcare providers managing obese children with difficult to control asthma. Recognizing and identifying an asthmatic patient is not always easy and a detailed medical history of the patient, with particular attention paid to their presenting and past symptoms, and a complete physical examination play pivotal and fundamental roles in determining the final diagnosis.

## 1. Introduction

Obesity and asthma are two of the most significant pediatric health problems worldwide, particularly in industrialized nations, and in recent decades their prevalence has increased dramatically. Between 1975 and 2016, the average age-adjusted body mass index (BMI) for children and adolescents around the world increased every decade by approximately 0.32 kg/m^2^ for girls and 0.40 kg/m^2^ for boys [1].

The rate of childhood obesity is steadily increasing, and the COVID-19 pandemic has made the issue even worse. According to a recent comprehensive meta-analysis involving 9 million children and teens, the number of cases of severe pediatric obesity has risen by 1.7 times since the early 2000 s [2].

Obesity in children aged 2 years and older is defined as a Body Mass Index (BMI) at or above the 95th percentile for their age and sex. To help reduce stigma and better align with medical practice, the terminology has shifted away from labels like “severe” or “morbid” obesity. Instead, the condition is now categorized into Obesity Grades 1, 2, and 3. These classifications are based on BMI percentiles using standardized growth charts such as those developed by the Centers for Disease Control and Prevention (CDC) or the World Health Organization (WHO). For children under 2 years of age, the CDC recommends using WHO weight-for-length growth standards, which are specific to age and sex, rather than BMI [3]. As metabolic conditions like diabetes, dyslipidemia, and nonalcoholic fatty liver disease—typically seen in adults—are increasingly identified in children, there is a growing push to shift the assessment of disease risk from adulthood to earlier in life for timely detection. Despite this shift, there is still no clear consensus on how to define metabolic diseases in children. To address this, researchers have suggested various indicators, including the Pediatric Simple Metabolic Syndrome Score, the Continuous Metabolic Syndrome Score, the Single-Point Insulin Sensitivity Estimator, and the Fatty Liver Index. These tools show promise for detecting metabolic issues early in children with obesity, though more validations are needed. Additionally, the approach to assessing obesity is evolving—from focusing solely on appearance to considering metabolic health and body composition. One emerging concept is sarcopenic obesity, which looks at the balance between muscle and fat. This measure has been applied to children and adolescents and is linked to their overall metabolic health [4]. Since 2015, the Centers for Disease Control and Prevention (CDC) has listed obesity as a major risk factor for asthma in children. The interrelationship between obesity and asthma derives from a complex interplay of biological, physiological, and environmental factors. The mechanisms leading to these disorders may start at an early age and include changes in lung mechanics, comorbidities, poor diet, low physical activity, alterations in insulin and/or glucose metabolism, and systemic inflammation. Among children who already have asthma, obesity is associated with worse disease severity, more frequent and severe exacerbations, poor symptom control, lower asthma-related quality of life, and higher risk of requiring intubation and mechanical ventilation [5].

## 2. Epidemiology

Several longitudinal epidemiological studies have reported that obesity is a risk factor for asthma later in life, as well as for worse asthma outcomes. Worldwide, there are more than 40 million overweight or obese children below the age of five. The prevalence of asthma in the European Union is 9.4% in children. According to the latest studies, allergic asthma, defined as asthma associated with sensitization to aeroallergens, is greatest in Northern and Western Europe, whereas the highest volume of bronchial asthma, as a chronic inflammatory respiratory condition characterized by dyspnea, coughing, and wheezing, occurs in Western and Eastern Europe [6]. According to self-reported data from the European Health Interview Survey, one in twenty-six individuals in the Slovak Republic have asthma. The National Register of Asthma in Slovakia further reports that 31% of asthma patients experience moderate or severe persistent asthma, while 27% have seasonal asthma symptoms [7]. In Italy, asthma is common among children, affecting approximately 10% of the pediatric population [8]. The Developmental Origins of Health and Disease (DOHaD) hypothesis suggests that early-life exposure to certain environmental factors can influence an individual’s health both during childhood and later in life. Asthma may start in utero too, which may be connected to high maternal BMI during pregnancy [9]. As a general trend, asthma prevalence increases as children’s body mass index percentile increases.

## 3. Asthma Phenotypes

Patients with both obesity and asthma can be classified into two primary categories: asthma complicated by obesity and asthma resulting from obesity (Table 1). The phenotype of asthma complicated by obesity includes all asthma phenotypes found in lean patients. A prominent phenotype is early-onset allergic asthma complicated by obesity. This type of asthma typically develops before the age of 12 and is characterized by elevated markers of allergic inflammation, including atopy, allergic symptoms, and high serum immunoglobulin E levels. Additionally, it involves eosinophilic infiltration of the airways and elevated exhaled nitric oxide levels, along with significant physiological changes, severe disease, and poor asthma control. These patients are prone to gain weight more rapidly than those with later-onset disease. Obesity may complicate the situation in this group, with both male and female patients equally affected [10,11]. The asthma resulting from obesity phenotype includes patients with later-onset disease (≥12 years old), who experience less allergic inflammation and are more likely to be female. Their asthma is typically characterized by less airflow obstruction and hyper-responsiveness, and it tends to be less severe compared to those with earlier-onset disease. Some patients show signs of neutrophilic airway inflammation, while others exhibit minimal cellular airway inflammation. Overall, they have better asthma control and lower symptom scores compared to patients with early-onset asthma. This late-onset phenotype has a Th2-low profile, with predominant neutrophil infiltration as well as low IgE and low eosinophilic infiltration. The “obese asthma” phenotype is complex and multifactorial, marked by additional symptoms, worse control, more frequent and severe acute episodes, reduced response to inhaled corticosteroids associated with a dose-dependent risk of increasing BMI trajectories over time, and lower quality of life compared to other asthma phenotypes [12].

Another factor contributing to obese asthma may be the adverse effects associated with systemic glucocorticosteroid therapy. Prolonged use of systemic glucocorticosteroids can lead to increased fat accumulation, particularly in the shoulders and the trunk region. Notably, the impact of these medications depends on both the dosage and the duration of treatment. Interestingly, in a recent study authors identified a potential protective effect of rescue medications against the development of overweight and obesity in asthmatic children. In a 10-year follow-up study involving over 2000 children, they found that those who regularly used rescue asthma medications had a reduced risk of becoming overweight or obese. The authors hypothesized that beta agonists might influence fat metabolism by acting on beta-2 adrenergic receptors found in adipocytes, thereby promoting lipolysis and offering a protective effect against obesity [13,14].

## 4. Pathophysiological Mechanisms

**Figure 1:**
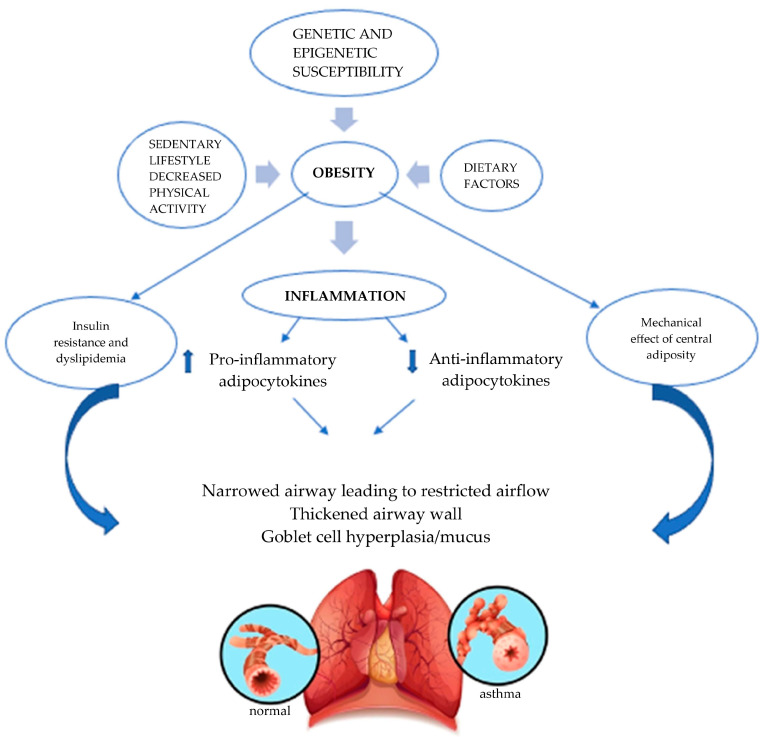
Factors associated with obesity-related asthma.

Obesity may worsen asthma through several mechanisms. These include the impact of excess body fat on lung function, which can be influenced by mechanical pressure from truncal fat and/or inflammation. Additionally, factors such as altered nutrient intake (both macro and micronutrients), a sedentary lifestyle, and associated obesogenic behaviors linked to obesity contribute to this worsening (Figure 1) [15]. The role of obesity in modulating the immune system, particularly through adipocytokines like leptin, has also been proposed as a key factor in the development of asthma in obese children. Asthma has also been linked to insulin resistance, dyslipidemia, and metabolic syndrome, all indicators of the metabolic dysregulation that occur in some but not all obese children [16]. Furthermore, genetic and epigenetic variations in molecules involved in metabolic regulation and associated inflammation have been identified as contributing factors in obesity-related asthma [17].

## 5. Changes in Lung Mechanics

Weight gain can significantly impact lung physiology, particularly by contributing to chest and abdominal adiposity, which can restrict lung expansion. One of the most notable effects of obesity on lung function is a decrease in functional residual capacity (FRC), with studies showing an inverse relationship between body mass index (BMI) and FRC. This reduction in FRC in obese individuals is mainly due to changes in the elastic properties of the chest wall. When lung volumes are low, the retractive forces of lung tissue on the airways are diminished, which may reduce the load on airway smooth muscle (ASM). This decreased load allows ASM to shorten more easily when activated, either due to an increase in parasympathetic tone or in response to bronchoconstrictor agents. Additionally, low tidal volume (VT) can further decrease strain on the ASM [5]. Obesity could be a risk factor for increased bronchial hyperresponsiveness. Both asthma and obesity contribute to impaired bronchodilation following deep inhalation, a mechanical airway dysfunction that, in asthmatic individuals, is linked to heightened airway inflammation. As lung volumes decrease, small airway caliber and flow reserves significantly diminish, which predisposes individuals to expiratory flow limitation (EFL). This results in dynamic gas trapping during increased ventilation, such as during exercise or bronchoconstriction episodes [18]. In general, childhood obesity is associated with normal or elevated forced expiratory volume (FEV1) and forced vital capacity (FVC), but reduced FEV1/FVC ratio. Recently, it has been observed that childhood obesity is linked to airway dysanapsis, a mismatch between the growth of lung parenchyma and airway caliber. This condition is reflected in normal or even supranormal FEV1 (greater than or equal to 21.645, which is the fifth percentile, or the lower limit of normal) and FVC (>0.674, or the 75th percentile). However, the larger effect on FVC leads to a lower FEV1/FVC ratio (around 80%) [19]. There are conflicting findings regarding patterns of fractional exhaled nitric oxide (FeNO), a marker of allergic airway inflammation, in obese children with asthma. One study found that higher adiposity was linked to greater asthma severity in children with low FeNO, whereas those with high FeNO experienced more severe asthma and poorer disease control. However, other studies reported no such association [20].

## 6. Immunology of Obesity-Related Asthma

Obesity is linked to ongoing low-level systemic inflammation, which can be viewed as a pro-inflammatory condition. Excess fat tissue plays a key role in the development of chronic, low-grade inflammation in individuals with obesity, leading to the release of cytokines. The number of macrophages in adipose tissue increases and they release various inflammatory molecules, such as tumor necrosis factor-α (TNF-α), interleukin 6 (IL-6), plasminogen activator inhibitor-1 (PAI-1), macrophage chemotactic protein-1 (MCP-1), and complement proteins. As a result, the circulating levels of these molecules are elevated in individuals with obesity [21]. Additionally, high-sensitivity C-reactive protein (hsCRP) levels are also found to be higher in obese patients. Obesity, as measured by BMI, has a significant correlation with hsCRP levels, while visceral fat is notably associated with IL-6 concentrations. There are various immune responses associated with obese asthma, including Type IVb, where T helper 2 cells play a central role, driven by cytokines such as interleukins 4, 5, 9, and 13. Type IVc, on the other hand, is marked by T helper 17 cells and Type 3 innate lymphoid cells that produce interleukin 17, which helps recruit neutrophils. Type V represents a dysregulated immune response, with heightened activation of T helper 1, 2, and 17 responses. Lastly, Type VI is associated with metabolic-induced immune dysregulation, commonly linked to obesity [22]. Asthma associated with obesity is mainly driven by non-eosinophilic mechanisms, particularly in the case of late-onset asthma [23]. Adipokines and other cytokines produced or induced by adipose tissue can also affect the lungs and airways. With increasing adiposity, there is a corresponding increase in leptin, which promotes oxidative and inflammatory responses from alveolar macrophages and a reduction in adiponectin and its protective effects [24]. All of these data favor the hypothesis that adipokines play important roles in the inflammatory pathogenesis of asthma in obese individuals.

**Figure 2:**
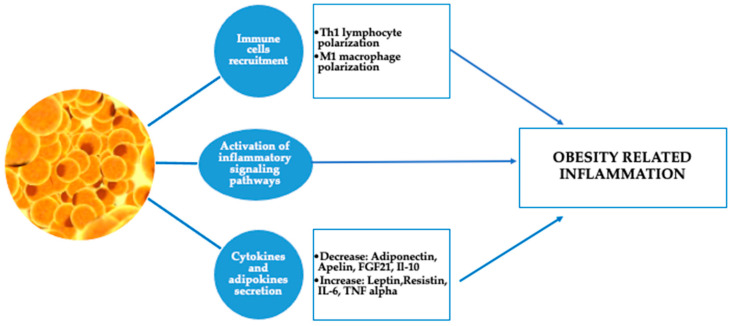
Adipose tissue and obesity-related inflammation. (T helper cell 1 (Th1), fibroblast growth factor 21 (FGF21), interleukin 10 (IL-10), interleukin 6 (IL-6), and tumor necrosis factor alpha (TNF alpha)).

Inhaled steroids may be less effective in overweight and obese individuals with asthma, potentially due to a systemic component of inflammation that extends beyond the airways. Peters-Golden et al. found that as BMI increased, the effectiveness of inhaled beclomethasone in improving asthma control days and reducing night-time awakenings declined. In contrast, the response to oral montelukast remained consistent regardless of BMI [25]. Obesity is part of metabolic syndrome, which is significantly involved in increasing the risk of a serious course of COVID-19 too, due to hyperdynamic circulation, as well as increased myocardial consumption of oxygen, which results in acute coronary events. “Cytokine storms” are also thought to lead to instability and rupture of atherosclerotic plaques, with subsequent thrombosis. Adipocytes and adipocyte-like cells (for example, lung lipofibroblasts), may play an important role in the pathogenesis of the response to COVID-19 [26]. Additionally, adipose tissue inflammation begins and persists due to the dysfunction of adipocytes, which release pro-inflammatory adipokines, and the infiltration of immune cells from the bone marrow that produce cytokines and chemokines. Although typically low-grade, this chronic inflammation can disrupt the function of distant organs and is believed to contribute directly to the complications associated with obesity (Figure 2) [27].

## 7. Oxidative Stress

Both asthma and obesity are linked to elevated oxidative stress, which refers to an imbalance between the production of pro-oxidants and the antioxidant defense mechanism in the body. This imbalance can trigger the release of pro-inflammatory cytokines and disrupt enzyme activity, exacerbating inflammation. This inflammation can activate immune cells like neutrophils and eosinophils, which generate reactive oxygen species (ROS). The rise in systemic oxidative stress may also correlate with increased production of lipid peroxidation products, protein carbonyls in plasma, and elevated plasma levels of isoprostanes, particularly isoprostane 8. Additionally, there is an increase in oxidized glutathione in bronchoalveolar lavage (BAL) fluid and an increase in nitric oxide (NO) levels in exhaled air. In children with asthma, higher levels of malondialdehyde (MDA) and lower levels of glutathione are found in exhaled breath condensate. Glutathione, an antioxidant, helps protect airway epithelial cells from free radicals, while MDA, a byproduct of ROS acting on membrane phospholipids, serves as a marker of oxidative stress. Asthma patients tend to have lower levels of superoxide dismutase (SOD) and catalase activity, which are associated with reduced lung function. Another obese asthma association is the change in nitric oxide (NO) metabolism. There is an inverse linear association between exhaled NO and increasing BMI in obese children [25].

## 8. Gender

Gender is a significant modifier of the obesity–asthma relationship. Boys are more susceptible than girls and it has been shown that boys who became obese at the age of 9–11 years have a two-fold greater risk of allergic asthma. Multiple studies have shown that the gender differences observed in the relationship between obesity and asthma appear to be linked to hormonal factors during the prepubertal stage (ages 9–11), particularly those related to pro-inflammatory hormones like leptin, adiponectin, and estrogen.

Increased BMI during adolescence is thought to be associated with higher estrogen levels, which could be a mechanism to explain female obesity and later-onset asthma [27].

## 9. Associated Comorbidities

Obesity-related diseases may increase the risk of developing asthma. Many studies have revealed that gastroesophageal reflux disease may worsen asthma by direct effects on airway responsiveness or via aspiration-induced inflammation, leading to bronchoconstriction. It has been reported that obesity is a risk factor for sleep-disordered breathing (SDB) conditions such as obstructive sleep apnea (OSA). Treating these disorders with nasal continuous positive airway pressure brings about significant improvement in asthma outcomes and peak expiratory flow (PEF) rates [24,28].

## 10. Macrometabolic and Micrometabolic Associations in Asthma

**Figure 3:**
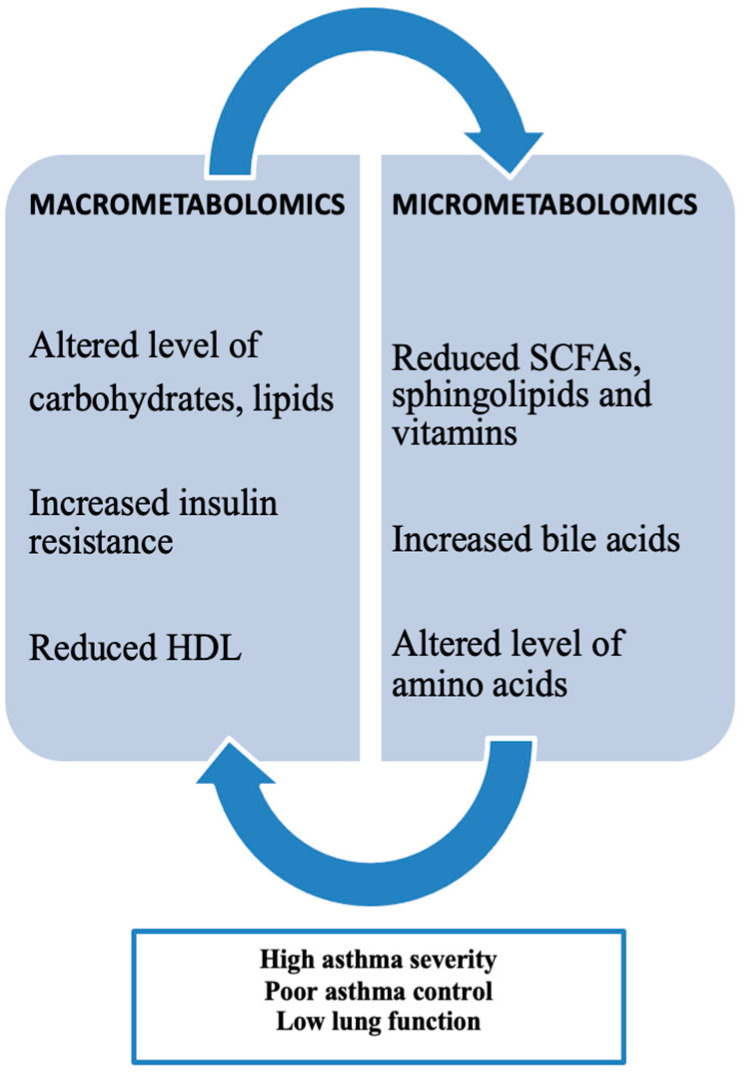
Macrometabolites, micrometabolites, and obesity-related asthma. (High-density lipoproteins (HDL) and short-chain fatty acids (SCFAs)).

Obesity-related asthma involves metabolic patterns categorized by both macrometabolic and micrometabolic factors in asthma, highlighting the important role of metabolomics (Figure 3).Current research indicates that metabolic disturbances are commonly observed in individuals with the “obese asthma” phenotype and align with those seen in metabolic syndrome. Metabolic dysregulation, such as insulin resistance (IR) and altered glucose metabolism, has been consistently linked to both childhood and adult asthma. Children with asthma tend to have a higher prevalence of IR compared to those without asthma, with IR levels correlating with proinflammatory markers like leptin and IL-6, which are associated with increased airway obstruction and reduced lung volumes. There is also evidence suggesting an inverse relationship between IR and lung function, as higher IR levels are linked to lower FEV1/FVC ratios. Children with asthma have a higher prevalence of dyslipidemia compared to non-asthmatic children, marked by lower high-density lipoprotein (HDL) and elevated low-density lipoprotein (LDL), total cholesterol, and triglyceride levels. Lower HDL levels are associated with lower FEV1/FVC ratios, further indicating an inverse relationship between dyslipidemia and pulmonary function. Beyond lipoproteins, free fatty acids (FFAs)—classified as small-chain, medium-chain, or long-chain based on their carbon chain lengths—play a role in the development of metabolic diseases like obesity, type II diabetes, and atherosclerosis, and have also been linked to respiratory diseases such as asthma [24].

Long-chain free fatty acids and their specific receptor FFAR1, which is expressed on airway smooth muscle, may serve as key modulators of airway smooth muscle tone and remodeling, potentially playing a critical role in linking obesity to asthma. A recent study highlights a novel role for gut microbes and their short-chain fatty acid (SCFA) metabolites in regulating blood pressure, having a benefit in reducing future cardiovascular risk in pediatric obese patients. Gut-derived SCFAs affect blood pressure and support nutrient absorption following a meal. Although the majority of nutrient absorption takes place in the small intestine, a considerable amount of carbohydrates—such as lactose, lactulose, and starches—undergo fermentation into SCFAs in the large intestine. SCFA concentrations rise in the blood vessels supplying the gut after a meal, triggering localized vasodilation that could enhance postprandial nutrient uptake [29]. N-3 long-chain fatty acids, such as DHA and EPA found in fish oil, have demonstrated anti-inflammatory effects in allergic diseases like asthma. Epidemiological and observational studies suggest that reduced intake of fish oil in modern diets may be linked to the rising prevalence of asthma [30]. Increased consumption of n-3 fatty acids has also been associated with lower incidences of asthma and fewer asthma-related symptoms. These fatty acids are metabolized into specialized pro-resolving mediators (SPMs)—including resolvins, protectins, and maresins—which help resolve airway inflammation by reducing eosinophilic infiltration and mucus production. Notably, the production of one such mediator (protectin D1) is impaired in individuals with severe asthma [31].

Various micrometabolic biomarkers have been linked to asthma. Sphingolipids are involved in cellular functions through their interactions at the cell membrane. Reduced sphingolipid synthesis has been associated with an increased risk of asthma. A key sphingolipid in asthma research is orosomucoid-like 3 (ORMDL3), which codes for a transmembrane protein located in the endoplasmic reticulum that regulates serine palmitoyltransferase (SPT) activity. Variations in the ORMLD3 gene have been linked to a heightened risk of asthma. Sphingolipids also help distinguish between non-allergic and allergic asthma in children, facilitating asthma endotyping. Studies have found that lower levels of sphingomyelins in infants at 6 months old are linked to a higher risk of developing asthma and wheezing by age 3. For children around 6 years old, reduced levels of certain phosphosphingolipids, such as sphinganine-1-phosphate, correlate with increased airway resistance. Amino acids also play a role in asthma development. The metabolism of L-citrulline is a significant biomarker in this context. In healthy individuals, the conversion of L-citrulline to L-arginine is sufficient, but asthma patients show decreased L-arginine production. L-arginine is essential for the production of nitric oxide (NO), a bronchodilator that helps prevent asthma progression. Disruptions in L-arginine metabolism can lead to nitric oxide deficiency and airway constriction, often due to increased arginase activity or the accumulation of inhibitors like asymmetric dimethylarginine (ADMA). Many studies have shown how patients with severe asthma with low or normal FeNO would have fewer exacerbations with L-arginine supplementation over a 3-month period compared with patients with high FeNO [24].

## 11. Diet and Nutrients

The low levels of vitamins in obese patients, which can be attributed to processed foods and changes in their eating habits, have direct impacts on micronutrient levels, including vitamins. Obesity is often linked to reduced circulating levels of vitamin D, which can result from factors such as limited sunlight exposure, lack of physical activity, insufficient intake of vitamin D-rich foods, dilution due to increased body volume, and storage in fat tissue. Additionally, as preadipocytes and adipocytes have receptors and play a role in vitamin D metabolism, it appears that insufficient vitamin D levels may contribute to adipogenesis and the development of obesity [32]. Authors have shown how in patients with bronchial asthma, the frequency of the appearance of symptoms during the day >2 times a week was higher in the group with deficiency and insufficiency of vitamin D in the blood, compared to the group with sufficient values of vitamin D in the blood (40% vs. 0%). All cases with sufficient values of vitamin D have manifested daily symptoms less than twice a week [33]. The data indicates that a loading dose of 50,000 IU of vitamin D, followed by a daily dose of 8000 IU for 16 weeks, is both safe and effective in reaching serum 25(OH)D levels of at least 40 ng/mL in children with obesity-related asthma. Among those receiving this regimen, 78.6% reached the target level [31,34]. Vitamin D deficiency was linked to decreased lung function in obese children with asthma, potentially serving as an indicator of both reduced airway function and lower lung volumes, regardless of systemic inflammation. Carotenoid levels are also associated with obese asthma. Clinical studies have shown that serum levels of total carotenoids were lowest in obese asthmatic adolescents. Furthermore, there was a direct positive association between total carotenoids and HDL, suggesting carotenoids could possibly be protective against metabolic dysregulation in pediatric obesity [24].

## 12. Obesity-Related Asthma and Gut Microbiota

Obesogenic diets high in fat and low in fiber modify the gut microbiome, promoting obesity and the development of allergic airway disease. The gut microbiota primarily acts as a biological barrier and plays a role in immune regulation. However, obesity can disrupt this balance, causing gut microbiota dysbiosis, reduced bacterial diversity, and an increase in firmicutes, while decreasing Bacteroidetes. These changes are key contributors to an impaired gut barrier and immune function. Research has shown that early reduction in gut microbial diversity can be a predictor of asthma development and may lead to allergies through an imbalance in Th2/Th1 immune responses. Obesity-related inflammation and gut dysbiosis can increase intestinal mucosal permeability, allowing lipopolysaccharides to enter the bloodstream. This triggers endotoxemia, where lipopolysaccharides bind to TLR4, activating the NF-kB pathway and leading to production of cytokines like TNF-a and IL-6. These cytokines can affect the lungs, potentially exacerbating asthma. Additionally, obesity-induced gut dysbiosis can disrupt cholesterol metabolism and lower intestinal bile acid levels, weakening the inhibitory effect of NLRP3. NLRP3 activation promotes IL-1b secretion through M1 macrophages, which in turn trigger AHR, a key feature of asthma. Bacterial colonization has an important role in fermentation of dietary fiber and generation of short-chain fatty acids (SCFAs) and low fiber diets may lead to changes in gut microbiome and SCFA levels, especially levels of SCFA propionate. Bacteroidetes bacteria, a major producer of SCFAs, are reduced in the gut in obesity and in the lungs of asthmatic patients [32]. Furthermore, in obese children with pediatric fatty liver disease, research has demonstrated how the gut microbiota and its metabolites influence the liver through the portal vein to regulate bile acid production as well as hepatic glucose and lipid metabolism. Disruptions in the gut–liver axis are crucial in the disease’s development, including alterations in the intestinal microbiome and liver barrier dysfunction. Modifying the gut microbiome has shown promising results in treating various metabolic disorders in experimental models, with the use of probiotics leading to reductions in intrahepatic triacylglycerol levels and AST activity [35]. Another influencing factor could be the effect of antibiotics on the microbiome. Antibiotic exposure early in life has been associated with asthma, obesity, and later-life atopy, modifying the maturation of the immune system. Probiotic supplementation in early life reduces the risk of atopy. An important point to discuss is the impact of lifestyle factors on both asthmatic and obese children. They can present with low levels of physical activity and generally sedentary behavior (e.g., watching television or playing video games), that may worsen lung function and physical performance.

## 13. Prenatal Factors

Prenatal factors may contribute to the pathogenesis of asthma in childhood. Prenatal diet and intrauterine exposure to pollutants and smoking can influence the development of an infant’s innate and adaptive immune systems. This can make the infant more vulnerable to early-life infections such as respiratory syncytial virus (RSV) and human rhinovirus (HRV), which can increase the risk of recurrent wheezing and asthma in later childhood [36]. Additionally, the 1000 days between pregnancy and a child’s second birthday are the most critical time for a child’s cognitive, physical, and social development. The right lifestyle for the child and surrounding environment during this time can have a profound impact on the child’s growth and development and reduce disease risk in the years to follow. On the other hand, among the most cost-effective preventive interventions is the promotion of breastfeeding; the benefits of breastfeeding for the infant include protection against overweight and diabetes, with long-term positive effects on later life [37].

## 14. Genetics and Genomics

Asthma and obesity share genetic factors. A group of genes increases the susceptibility to both asthma and obesity. Chromosome 5q contains the genes ADRB2 and beta-2 adrenergic receptor gene, which influence sympathetic nervous system activity and regulate airway tone and resting metabolic rate. The Agr16 polymorphism of this receptor is linked to certain asthma phenotypes, including nocturnal asthma. The Gln27 polymorphism of the same receptor is highly associated with obesity and influences the bronchodilator response to beta-2 agonists. Chromosome 5q also contains the gene NR3C1, which codes for glucocorticoid receptors, and its polymorphism is significantly associated with bronchial asthma and obesity, influencing the accumulation of abdominal visceral fat. Several studies have shown a link between the 308-G/A polymorphism in the TNF-alpha gene and both asthma and obesity. The NcoI variant in the lymphotoxin-A gene (LTA, located at 6p21.3), which interacts with the TNF 308-G/A polymorphism, has been connected to various asthma-related traits, including atopic asthma. Meanwhile, the T60N polymorphism of the LTA gene has been associated with waist circumference and other metabolic syndrome characteristics. Chromosome 12q, which contains genes for inflammatory cytokines, also plays a role in both asthma and obesity. Several variants of the vitamin D receptor gene (VDR, located at 12q13), which regulate immune functions, can suppress Th2-mediated allergic airway diseases. For instance, in Italian children, lower serum vitamin D levels correlate with poorer lung function, increased exercise-induced hyperresponsiveness, and worse asthma control. Furthermore, a recent genome-wide association study (GWAS) identified DENND1B as an asthma susceptibility gene in children, with evidence suggesting that certain variants of DENND1B may also be linked to BMI in children with asthma.

However, the association has not been replicated in independent data sets and the heterogeneous effect of DENND1B points to complex associations with the studied diseases that deserve further investigation [38].

## 15. Diagnosis

The diagnosis of asthma is primarily based on comprehensive patient history, which helps identify the pattern of symptoms (episodic or continuous), triggers (such as work, exercise, exposure to allergens, or smoking), and the timing of onset in relation to obesity and asthma. Spirometry is used to assess if there is baseline airflow limitation (obstruction), characterized by a reduced FEV1/FVC ratio, determine the reversibility of this obstruction, and rule out restrictive patterns as an alternative cause of dyspnea [39]. Bronchoprovocation testing is another valuable diagnostic tool, particularly for patients with unusual asthma presentations or isolated symptoms like a persistent cough. Peak expiratory flow (PEF) is more commonly used to monitor patients with a confirmed asthma diagnosis or evaluate the impact of specific occupational or environmental triggers, rather than being used for initial asthma diagnosis [40]. Measuring nitric oxide in exhaled breath can also assist in diagnosing asthma. Elevated levels of exhaled nitric oxide (fractional exhaled nitric oxide [FENO] ≥40–50 parts per billion) can help confirm asthma. In children, a low FENO is associated with higher adiposity indicators (such as BMI, body fat percentage, and waist circumference), while a high FENO suggests poorly controlled asthma [41]. While there are no specific blood tests for asthma, a complete blood count (CBC) with a differential white blood cell analysis can help detect eosinophilia or anemia, which might be relevant in certain cases. Allergy testing includes measuring the total serum immunoglobulin E (IgE) levels and testing for specific IgE antibodies against inhalant allergens, either through blood tests or skin testing.

## 16. Management of Childhood Obese Asthma

**Figure 4:**
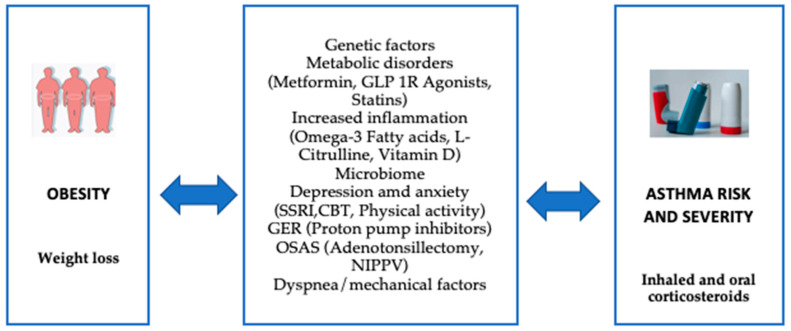
Selected pathways involved in obesity-related asthma and potential interventions for the management of patients with asthma in the setting of obesity. (Glucagon-like peptide-1 receptor (GLP-1R), selective serotonin reuptake inhibitor (SSRI), Cognitive behavioral therapy (CBT), gastroesophageal reflux (GER), Obstructive Sleep Apnea Syndrome (OSAS), Noninvasive Positive Pressure Ventilation (NIPPV)).

Managing obese children with asthma requires special attention and is more challenging than managing asthmatic children with normal weight. Key differences may include reduced responsiveness to standard controller medications, varying thresholds for determining asthma phenotypes to guide biological therapy, the significance of comorbidities, and the importance of weight loss (Figure 4). Research has shown that weight reduction through intensive lifestyle changes, such as modifications in diet, physical activity, and behavior, can have a significant impact on children with both obesity and asthma. Involving the whole family in these changes is essential. Approaches like motivational interviewing, goal-setting encouragement, behavior monitoring, and positive reinforcement appear to be effective strategies, helping clinicians personalize interventions based on the patient’s behavioral and psychological needs. Generally, recommendations include a diet rich in fruits and vegetables, limiting sugary, sweetened drinks, regular breakfasts, allowing the child to have some control over their meals, avoiding overly restrictive feeding practices, and encouraging the involvement of the entire family in making lifestyle changes [17].

A high-fiber diet increases SCFA concentrations in the gut and circulation, thereby suppressing weight gain and allergic inflammation, which are dependent on gut microbiota metabolism. Studies have also shown a link between obesity and changes in fecal short-chain fatty acid (SCFA) profiles. Increasing SCFA levels could therefore be helpful for individuals with obesity-related asthma. Additionally, gut bacteria can ferment undigested or unabsorbed food to produce conjugated linoleic acid (CLA), including trans-10 and cis-12 CLA, which may help reduce overall body fat by targeting fat stores in areas such as visceral, inguinal, and brown/interscapular fat. This suggests that CLA supplementation might be more effective in preventing fat mass regain after weight loss than in promoting weight loss itself. Certain bacteria, like *Bifidobacterium*, *Bifidobacterium pseudolongum*, and *Bifidobacterium breve*, can convert free linoleic acid and alpha-linolenic acid into various forms of CLA. As a result, CLA and related compounds present a promising new approach for weight management that could also alleviate symptoms of obesity-related asthma [32].

Many studies have shown that vitamin D supplementation in children with mild asthma, and achieving vitamin D levels of 30 ng/mL, may be a useful strategy for optimizing lung function and a way of remodeling patients previously diagnosed with bronchial asthma [33]. Studies have shown the beneficial effect of statins in the context of asthma, lowering FFAs and subsequently decreasing airway hyperresponsiveness by modulation of ASM cells. Innovative research has discovered anti-inflammatory and immunomodulatory properties of statins beyond their cholesterol lowering function, demonstrating that simvastatin attenuates eosinophilic airway inflammation via inhibition of HMG-CoA [22]. Drug therapy for obesity, such as orlistat, liraglutide, and other options, may be suitable for patients who have not achieved weight loss through behavioral lifestyle changes alone and are experiencing serious obesity-related complications [42]. Bariatric surgery is generally recommended for obese asthma patients who have a BMI of 35 kg/m^2^ or higher and who have not been able to lose sufficient weight by other methods [43].

## 17. Pharmacological Treatment of Obese Asthma

Pharmacological treatment is the mainstay of management in most patients with asthma, though in obese patients, treatment of comorbidities and lifestyle interventions are likely to be particularly important. The Global Initiative for Asthma (GINA) acknowledges the potential role of obesity in both the diagnosis and management of asthma. However, it is crucial to recognize that some respiratory symptoms commonly seen in obese individuals, such as shortness of breath during physical activity, might resemble asthma symptoms [44]. As a result, in obese patients, asthma diagnosis should involve objective tests to assess variable airflow limitations that respond to bronchodilators.

Children with both obesity and asthma might show reduced response to asthma medications, but inhaled corticosteroids (ICS) and bronchodilators remain the primary treatment options. Both GINA and other national guidelines, including those in the US, suggest that clinicians should consider weight loss interventions for obese asthma patients [45]. Patients with severe asthma who require high doses of inhaled glucocorticoids along with a second controller or continuous oral glucocorticoid treatment may benefit from monoclonal antibodies against IL-5 (mepolizumab and reslizumab), IL-5 receptor alpha (benralizumab), and the IL-4 receptor alpha subunit (dupilumab) [46]. Long-term systemic glucocorticoid use is generally avoided due to side effects like weight gain and metabolic dysfunction. For these patients, treatment options like omalizumab are considered, especially in those weighing over 150 kg [46,47]. The primary aim in asthma management is to optimize symptom control, reduce the risk of exacerbation, and minimize medication side effects, enabling patients to participate in work, school, recreation, and sports without restrictions. The four key components of asthma management are patient education, managing triggers, monitoring changes in symptoms or lung function, and pharmacological treatment.

## 18. Conclusions

Obesity is an important risk factor for asthma morbidity in children. More than simple co-existence links asthma and obesity. Bi-directional pathophysiology links have been explored but causality is not clear. Possible underlying mechanisms may involve a common genetic factor, dietary and nutritional influences, changes in the gut microbiome, systemic inflammation, metabolic issues, and alterations in lung structure and function.

Systemic inflammation in obesity upregulating airway inflammation in asthma in combination with mechanical alterations in respiratory function play key roles. Both obesity and asthma lead to decreased physical activity resulting in further deterioration in the conditions as a vicious circle. Patient susceptibility depends on the interaction with genetic, environmental, developmental, lifestyle, and clinical factors. The obese asthma phenotype conveys more severe disease and poorer response to treatment. Weight management through multidisciplinary intervention and effective asthma management is a key strategy, including increasing physical activity and improving adherence to dietary guidelines. Currently, weight loss medications are allowed only in rare and very selected cases and bariatric surgery not recommended before 18 years of age.

## 19. Future Directions

Further research and understanding of the obesity–asthma relationship is needed, enhancing endotyping and phenotyping of children with obesity-related asthma, identifying reliable biomarkers, designing pediatric-specific studies that consider important stages of growth and development, and guiding clinical management and interventions. It is crucial to provide high-risk children and adolescents with strategies that encourage physical activity and healthy habits, while also strengthening asthma self-management and treatment adherence, in order to reduce long-term morbidity and mortality.

The development of hand-pocket tools—like electronic monitoring devices and mobile apps—can help improve communication with young patients dealing with asthma and obesity. Features such as medication reminders support better treatment adherence, while apps and serious games can enhance understanding of the condition. These tools also encourage proactive management of asthma, leading to earlier interventions and potentially lowering healthcare costs over time. Early interventions for children with asthma and/or wheezing may be warranted to prevent a vicious cycle of worsening obesity and asthma that could contribute to the development of other metabolic diseases in later life.

## Figures and Tables

**Table 1:** Obese asthma phenotypes.

Obese Asthma Phenotypes	Early-Onset Asthma	Late-Onset Asthma
Age at onset	<12 years	>12 years
Gender	Female = Male	Female > Male
Airway function (FEV1, FVC)	Severe decrease in airway function	Minimal airway obstruction
Nature	Atopic	Non-atopic
Airway hyperreactivity	Severe airway hyperresponsiveness	Less airway hyperresponsiveness
Symptom score	High symptom score	Low symptom score
Airway inflammation	Eosinophilic airway infiltration	Neutrophilic infiltration
Th1-Th2 profile	High Th2 biomarkers	Low Th2 biomarkers
